# Pharmacokinetic/pharmacodynamic integration and modelling of florfenicol for the pig pneumonia pathogens *Actinobacillus pleuropneumoniae* and *Pasteurella multocida*

**DOI:** 10.1371/journal.pone.0177568

**Published:** 2017-05-26

**Authors:** Lucy Dorey, Ludovic Pelligand, Zhangrui Cheng, Peter Lees

**Affiliations:** Comparative Biological Sciences, Royal Veterinary College, London University, London, United Kingdom; Universidad de Cordoba, SPAIN

## Abstract

Pharmacokinetic-pharmacodynamic (PK/PD) integration and modelling were used to predict dosage schedules for florfenicol for two pig pneumonia pathogens, *Actinobacillus pleuropneumoniae* and *Pasteurella multocida*. Pharmacokinetic data were pooled for two bioequivalent products, pioneer and generic formulations, administered intramuscularly to pigs at a dose rate of 15 mg/kg. Antibacterial potency was determined *in vitro* as minimum inhibitory concentration (MIC) and Mutant Prevention Concentration in broth and pig serum, for six isolates of each organism. For both organisms and for both serum and broth MICs, average concentration:MIC ratios over 48 h were similar and exceeded 2.5:1 and times greater than MIC exceeded 35 h. From *in vitro* time-kill curves, PK/PD modelling established serum breakpoint values for the index AUC_24h_/MIC for three levels of inhibition of growth, bacteriostasis and 3 and 4log_10_ reductions in bacterial count; means were 25.7, 40.2 and 47.0 h, respectively, for *P*. *multocida* and 24.6, 43.8 and 58.6 h for *A*. *pleuropneumoniae*. Using these PK and PD data, together with literature MIC distributions, doses for each pathogen were predicted for: (1) bacteriostatic and bactericidal levels of kill; (2) for 50 and 90% target attainment rates (TAR); and (3) for single dosing and daily dosing at steady state. Monte Carlo simulations for 90% TAR predicted single doses to achieve bacteriostatic and bactericidal actions over 48 h of 14.4 and 22.2 mg/kg (*P*. *multocida*) and 44.7 and 86.6 mg/kg (*A*. *pleuropneumoniae*). For daily doses at steady state, and 90% TAR bacteriostatic and bactericidal actions, dosages of 6.2 and 9.6 mg/kg (*P*. *multocida*) and 18.2 and 35.2 mg/kg (*A*. *pleuropneumoniae*) were required. PK/PD integration and modelling approaches to dose determination indicate the possibility of tailoring dose to a range of end-points.

## Introduction

In recent years there have been major advances in designing dosage schedules of antimicrobial drugs, through integration and modelling of pharmacodynamic (PD) and pharmacokinetic (PK) data. These approaches have improved strategies for predicting drug dosages that optimise efficacy and minimise opportunities for emergence of resistance [1–7].

The most commonly used PD parameter to define potency of antimicrobial drugs is minimum inhibitory concentration (MIC), the lowest concentration that inhibits visible bacterial growth after 24 h incubation under standard conditions [8]. Also increasingly used is mutant prevention concentration (MPC), the concentration preventing the growth of the least susceptible cells in high-density bacterial populations [9]. Integration of *in vitro* generated potency indices with *in vivo* generated PK data has been widely used to generate three indices, the ratios maximum plasma/serum concentration (C_max_)/MIC and area under plasma/serum concentration-time curve (AUC_24h_)/MIC and the time for which concentration exceeds MIC, T>MIC.

Integrated PK/PD data are commonly supplemented by time-kill studies to provide information on the speed of onset and time course of antimicrobial effect, enabling classification of drugs as time-, concentration- or co-dependent in their killing actions. Moreover, numerical values for PK/PD breakpoint indices can be determined by PK/PD modelling of data from time-kill studies [10–11]. As well as generating PK/PD breakpoints, these PK and PD data may be used, with wild type MIC distributions, to estimate optimal doses to provide pre-determined levels of kill, whilst minimising emergence of resistance. Ideally, predicted doses are then correlated with clinical and bacteriological cures in animal models and clinical trials. As discussed by Giraudel et al. [12], Lees et al. [13] and Toutain and Lees [14], dose determination through linking PK and PD data is generally preferred to dose titration studies, in which the body may be regarded as a black box; the doses are administered and responses measured but nothing is known of the plasma concentration-effect relationship and estimated dosages may be effective but are unlikely to be optimal.

**Fig 1:**
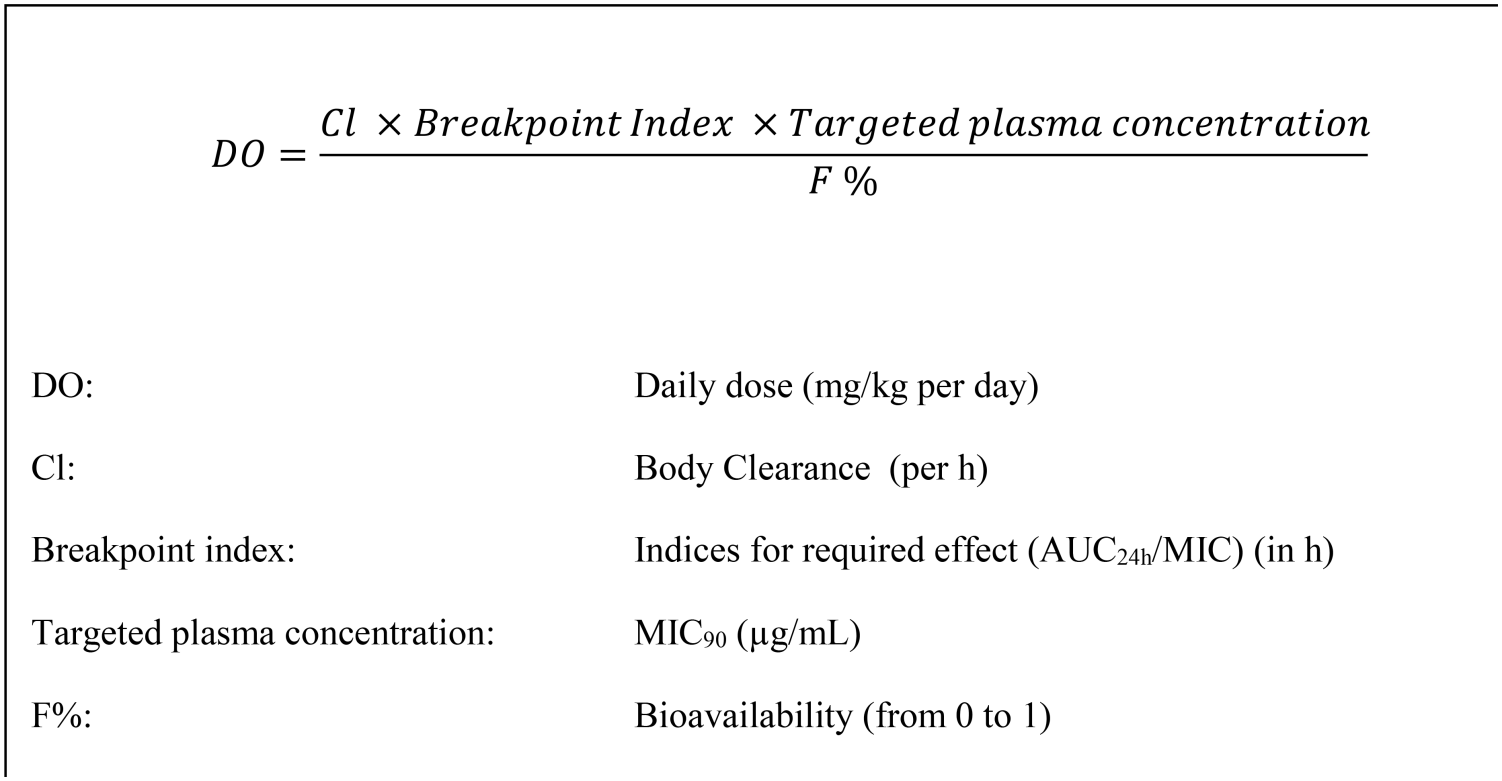
Formula for calculation of daily dose. Calculation at steady state based on pharmacokinetic and pharmacodynamic variables.

The PK/PD approach to dose prediction for amoxicillin for treatment of calf pneumonia caused by the pathogens *Mannheimia haemolytica* and *Pasteurella multocida* was described by Lees et al. [15]. The present study has adopted similar methods to estimate dosages of florfenicol for the pig pathogens *Actinobacillus pleuropneumoniae* and *P*. *multocida*. Fig 1 presents the equation used to calculate daily dosage, when steady-state has been reached.

The aims of this study were: (1) to determine the plasma concentration—time profile for florfenicol administered to pigs intramuscularly at the recommended dose rate of 15 mg/kg and derive PK variables by non-compartmental modelling; (2) to integrate *in vivo* PK variables with *in vitro* PD indices of potency (MIC and MPC) to obtain values of C_max_/MIC, C_max_/MPC, T>MIC, T>MPC and ratios of average concentrations (C_av_)/MIC and C_av_/MPC for *A*. *pleuropneumoniae* and *P*. *multocida*; (3) to model data from time-kill studies of *A*. *pleuropneumoniae* and *P*. *multocida* in order to generate PK/PD breakpoint values of AUC_24h_/MIC for three levels of bacterial kill, bacteriostasis, bactericidal and 4log_10_ reduction in inoculum count; (4) to use PK, PD, serum protein binding data and literature MIC distributions in Monte Carlo simulations to estimate dose schedules required for: (a) bacteriostatic and bactericidal levels of kill; (b) for 50 and 90% target attainment rates (TAR); and (c) for single dosing and for daily dosing at steady state.

## Materials and methods

### Origin, storage and selection of bacterial isolates

Twenty isolates of *P*. *multocida* were supplied by Don Whitley Scientific (Shipley, West Yorkshire, UK). They also supplied three ATCC reference strains, *A*. *pleuropneumoniae* ATCC 27090^®^, *Enterococcus faecalis* ATCC 29212^®^ and *Escherichia coli* ATCC 25922^®^ to validate MIC determinations. Eight isolates of *A*. *pleuropneumoniae* were supplied by A. Rycroft (Royal Veterinary College, Hawkshead Campus, Hatfield, Herts., UK). All *P*. *multocida* and *A*. *pleuropneumoniae* isolates were derived from EU field cases of pig pneumonia. Based on three criteria, six isolates of each species were selected: (1) ability to grow logarithmically in both broth and pig serum; (2) susceptibility to florfenicol in disk diffusion assays (data not shown); and (3) the highest and lowest broth MICs and four isolates with intermediate MICs, determined using two-fold dilutions (data not shown). This initial selection procedure ensured that all isolates could be used in subsequent investigations in both growth media and that they comprised a small but diverse range of susceptible isolates.

### Determination of minimum inhibitory and mutant prevention concentrations

Bacterial isolates for in vitro studies were supplied by Don Whitley Scientific (*A*. *pleuropneumoniae*) and the Royal Veterinary College (*P*. *multocida*) [16]. All were clinical isolates of European (UK, France and Germany) origin [16]. MICs and MPCs were determined by microdilution and agar method, respectively, for six isolates each of *A*. *pleuropneumoniae* and *P*. *multocida*, MIC in accordance with CLSI guidelines [8] and MPC as described by Dorey et al. [16], using artificial broths, Cation Adjusted Mueller Hinton Broth (CAMHB) for *P*. *multocida* and Columbia Broth supplemented with NAD (CB) for *A*. *pleuropneumoniae*. In addition, the methods were adapted using porcine serum supplied by Invitrogen Gibco (Porcine Serum, ThermoFisher, Paisley, UK) in place of broths to enable comparison of potency in the two matrices. To improve accuracy of estimates for each isolate, five sets of overlapping 2-fold serial dilutions of florfenicol were prepared in 96 well plates. Methods for MIC, MBC and MPC were as described and reported by Dorey et al. [16]. Each test on each isolate was repeated three times.

For MPC determinations, fresh cultures were grown on agar and approximately 100 single CFUs collected on a sterile swab to inoculate culture from plates to a volumetric flask containing 200 mL of pre-warmed broth. This was placed in a static incubator overnight. Next day, 1 mL of culture was added to 9 mL CAMHB and placed in an orbital incubator for 4 h at 37°C and 180 rpm. After 4 h, the bacterial suspension yields a theoretical count of 1–2 x 10^11^ CFU/mL. A spot plate was used to confirm inoculum density. Drug concentrations used were 1, 2, 4, 8, 16, 32, 64 and 128 multiples of the MIC for each isolate. The concentration ranges were narrowed down two further times. Florfenicol solution (0.5 mL volume) was applied to agar plates. After drying, 100 μL of culture were added and the plate allowed to dry. Plates were incubated at 37°C for 72 h and checked for growth every 24 h. MPC was the lowest florfenicol concentration inhibiting bacterial growth completely after 72 h incubation. Each experiment was repeated in triplicate for six isolates of each test organism. The method was validated using a parallel design against that described by Blondeau (2009) and yielded similar results for two isolates of *P*. *multocida* repeated three times. The method described here was equal to or within one doubling dilution of that from Blondeau method, in this case, 4–8 μg/mL (Dorey et al, 2016).

### Florfenicol pharmacokinetics

Individual animal plasma concentration-time profiles for florfenicol generated in young pigs were obtained for two florfenicol containing products (Nuflor, Merck Animal Health, New Jersey, U.S., and Norfenicol, Norbrook Laboratories, Newry, Northern Ireland). The pharmacokinetic studies were conducted under PPL 2626 (Drug Pharmacology in Domestic Species, effective 11/10/2007). This license underwent review by the Norbrook Laboratories Ethical Review Committee in October 2007. The study was Good Laboratory Practice compliant, conducted by Norbrook Laboratories and approved by the company’s Ethics Committee. All aspects of the study were conducted according to the requirements of the relevant Project License (PL2626) held by the Sponsor for conducting pharmacokinetic studies in the relevant species.

All pigs were Landrace Cross males and approximately 2 months of age. The animals were supplied from Ballyedmond Castle Farms Limited and were individually identified by ear tag and tag numbers recorded. They were housed individually in pens. Each animal was healthy, physiologically normal, and representative of European porcine stock and weighed between 32.5–52.0 kg. They were fed a cereal-based ration manufactured without the addition of antibiotics ad libitum. Drinking water was freely available throughout the animal phase of the study. The temperatures within the housing were measured daily using validated max/min thermometers. Individual daily health monitoring of the animals was conducted throughout the study. The animals were monitored for illness, injury and evidence of adverse reactions to treatment. Injection site monitoring was conducted for 3 days following test article administration, for reactions of swelling, hardness/softness, pain, heat and redness. No systemic adverse reactions to treatment were recorded. In addition no administration site reactions were noted following monitoring for 3 days after administration for reactions of swelling, hardness/softness, heat, redness/discolouration and pain.

Each product was administered intramuscularly at a dose rate of 15 mg/kg. For each product the theoretical doses were calculated based on the actual florfenicol concentration and the bodyweights of the animals one day before administration. Each dose was administered by intramuscular injection to the right neck. A 16G x 1 inch needle and appropriately sized syringes were used for administration. No analgesics were used during the study, first because they were unnecessary and second due to the potential interference with the pharmacokinetics of florfenicol. Blood samples were taken by venepuncture from the jugular vein into heparinised vacutainers. Immediately after collection, samples were placed on ice prior to centrifugation. After centrifugation, the plasma was sub-sampled into 3 appropriately labelled cryovials and stored at < -20°C then despatched to the testing facility, where they were stored at< -20°C until assayed. Following completion of the pharmacokinetic study, animals were returned to stock, i.e. animals were not killed at the end of the study, as they remained in good health.

As the two products had been shown to be bioequivalent, the data were pooled, to provide a richer data set of 34 animals for non-compartmental analysis (NCA) using the programme WinNonLin 6.4 (Pharsight Corporation, Mountain View, CA, USA). Variables calculated were C_max_, C_av_, AUC_0-last_, Area under first moment curve (AUMC_0-last_), time of maximum concentration (T_max_), clearance scaled by bioavailability (Cl/F) and terminal half-life (T_1/2_). The programme extrapolated the data to provide AUC_0-∞_ and three time periods were investigated (0–48 h, 0–24 h and 24-48h) for determination of average concentrations.

### PK/PD integration

PK/PD indices, calculated using individual animal PK values and mean MIC and MPC values, were: (1) C_max_/MIC, T>MIC and C_av_/MIC ratio for three time periods, 0–24 h, 24–48 h and 0–48 h; and (2) C_max_/MPC, T>MPC and C_av_/MPC ratio for three time periods, 0–24 h, 24–48 h and 0–48 h. T>MIC and T>MPC were determined using terminal lambda_z, obtained by NCA, using the equation C = A*exp(-b*t), where C is drug concentration, t is time, A is the concentration at T_last_ and b is lambda_z. At least three concentration-time points on the terminal phase were used for estimating lambda_z. For the prediction of drug concentrations in the terminal phase, the method was compared with the prediction using compartmental modelling; the former generated better fitting with significant smaller residuals.

### PK/PD modelling

For six isolates of each pathogen, *in vitro* growth inhibition curves were determined using eight multiples of MIC, as previously described [16]. Each test was repeated in triplicate for six isolates each of *A*. *pleuropneumoniae* and *P*. *multocida* in both artificial medium (CB and CAMHB, respectively) and serum. The maximum CFU/mL count was set to 1x10^10^ CFU/mL and the lower limit of quantification (LLOQ) was 33 CFU/mL.

**Fig 2:**
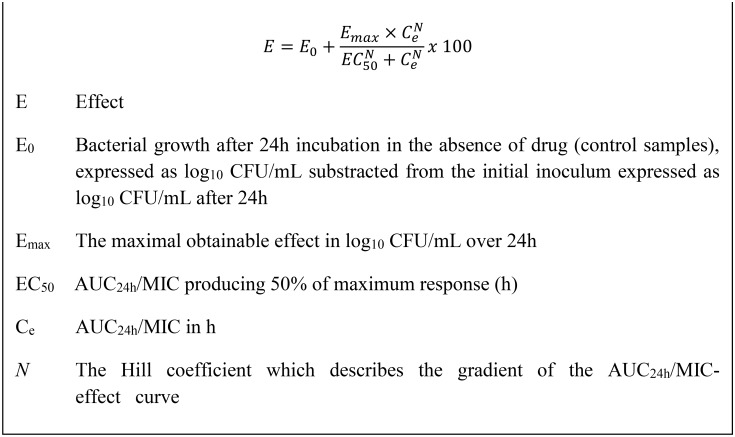
The sigmoidal Emax equation. Used to model time-kill data by non-linear regression [13].

**Fig 3:**
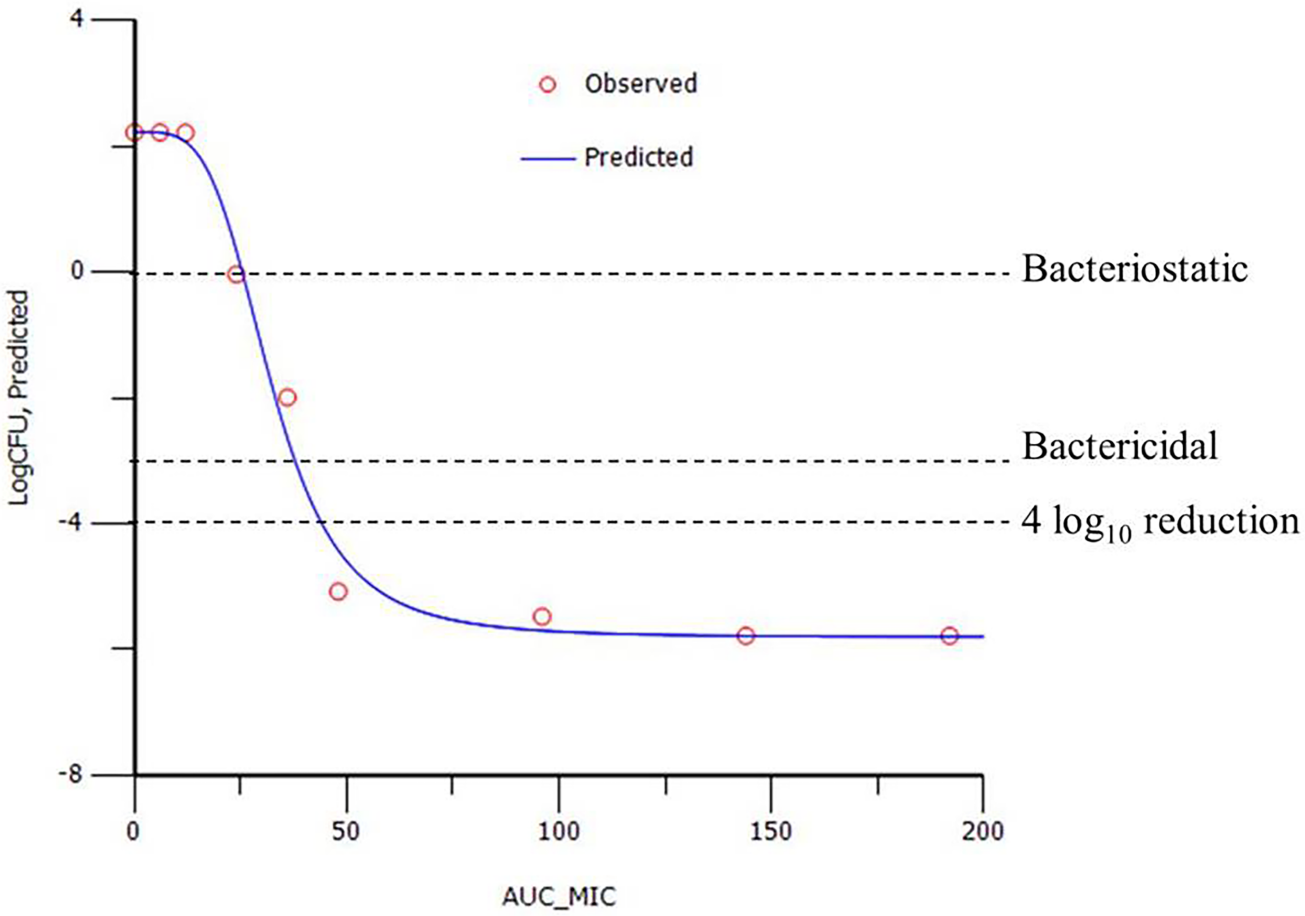
Example plot of AUC_24h_/MIC versus change in bacterial count from initial count (log_10_ CFU/mL). Obtained from in vitro time-kill data for florfenicol. Each point represents an experimental value. The curve is the line of best fit based on the sigmoidal Emax equation.

The sigmoidal E_max_ equation (Fig 2) was used to model AUC_24h_/MIC data from growth inhibition curves, using the non-linear regression programme WinNonLin to predict plots of log_10_ change in CFU/mL versus AUC_24h_/MIC (Fig 3). PK/PD breakpoints were determined for three levels of growth inhibition after 24 h incubation: E = 0, bacteriostatic, that is 0log_10_ reduction in CFU/mL; E = -3, bactericidal, 3log_10_ reduction in CFU/mL; and E = -4, 4log_10_ reduction in bacterial count.

### Dose determination using Monte Carlo simulations

**Fig 4:**
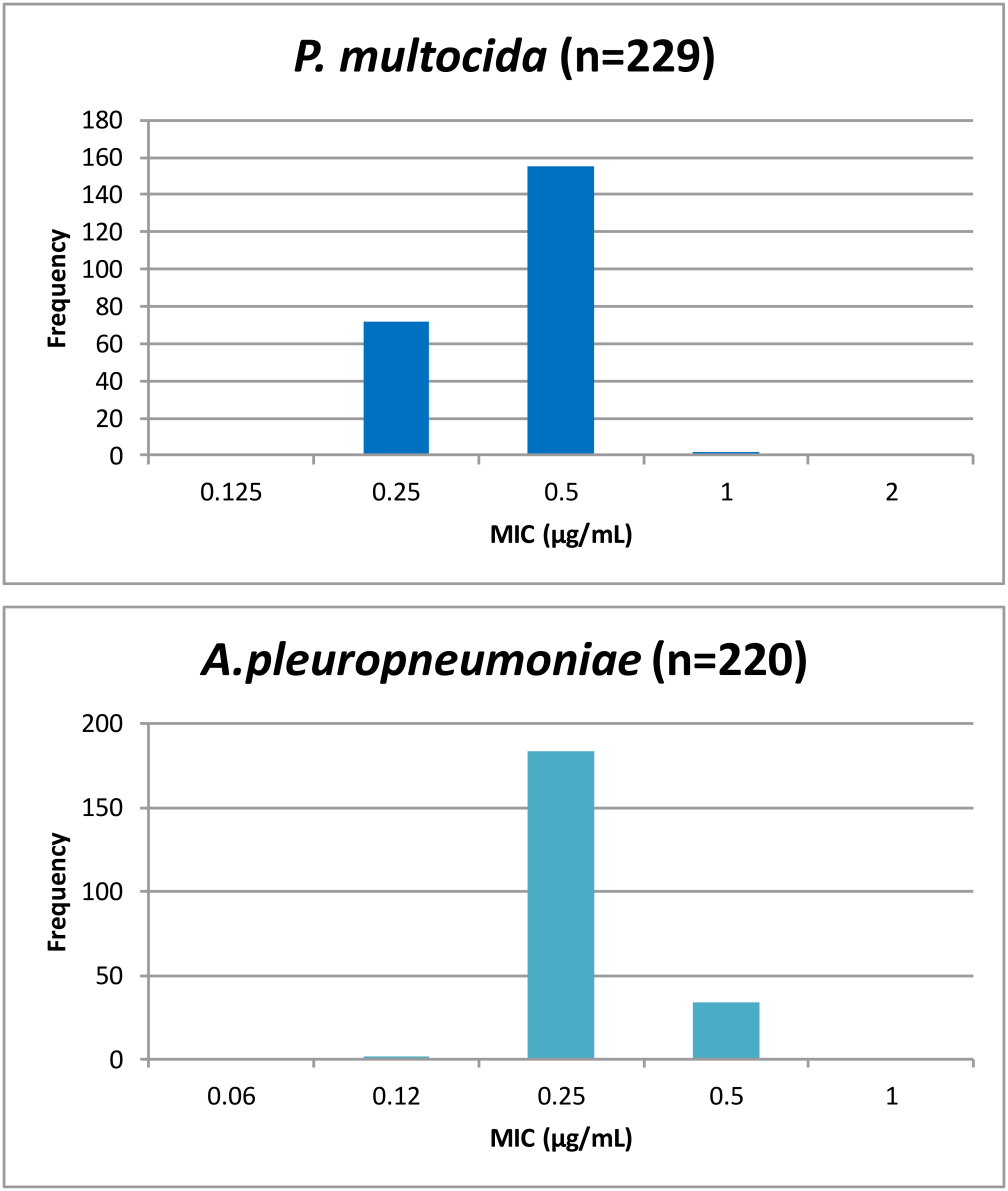
MIC distribution of *P*. *multocida* (n = 230) and *A*. *pleuropneumoniae* (n = 219).

**Fig 5:**
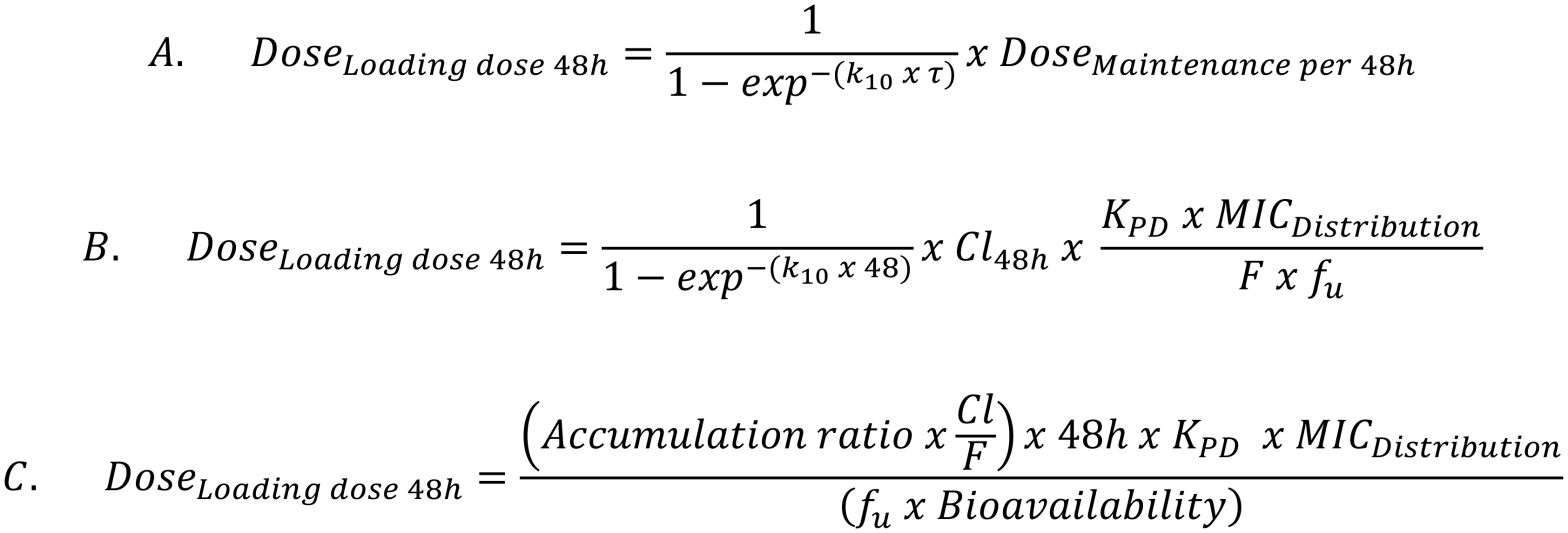
Formulae for calculation of the loading dose for 48h duration of action.

Monte Carlo simulations provided predictions of dose. Data input comprised: (1) whole body clearance scaled by bioavailability; (2) drug binding to serum protein; (3) AUC_24h_/MIC breakpoints derived from time-kill curves by PK/PD modelling; and (4) MIC field distribution data obtained from the scientific literature [17] (Fig 4). MIC_90_ was used for the deterministic approach and MIC distribution data were used for Monte Carlo simulations. Monte Carlo simulations predicted (Fig 5): daily dose at steady state; single doses for three time periods, 0–24, 0–48 and 0–72 h, as described by Lees et al. [15]. All dosages were computed using Monte Carlo simulations in Oracle Crystal Ball (Oracle Corporation, Redwood Shores, CA, USA) for target attainment rates (TAR) of 50 and 90%. For the MIC distributions, values were corrected using the serum:broth MIC ratios for each pathogen, as the reported MIC literature values were determined in broth. The probabilities of distribution for the dosage estimation were run for 50,000 simulated trials.

## Results

### Minimum inhibitory and mutant prevention concentrations

MICs and MPCs were determined for six isolates each of *P*. *multocida* and *A*. *pleuropneumoniae*, in broth and pig serum. Geometric mean MIC values (SD) for *P*. *multocida* were 0.42 (0.13) and 0.30 (0.16), respectively. Corresponding values for *A*. *pleuropneumoniae* were 0.38 (0.11) and 0.39 (0.17). Mean MPC values (SD) in broth and serum for *P*. *multocida* were 5.63 (1.70) and 4.08 (1.69) respectively. For *A*. *pleuropneumoniae*, MPC values were 3.84 (0.70) and 4.89 (1.93) respectively.

### Florfenicol pharmacokinetics

Pharmacokinetic variables were calculated by non-compartmental analysis from plasma concentration-time data for 34 pigs, comprising data for 17 animals receiving product A (Nuflor) and 17 pigs administered product B (Norfenicol). Each product was administered intramuscularly at a dose rate of 15 mg/kg and the two products were bioequivalent. Table 1 presents mean values.

**Table 1:** Pharmacokinetic variables (mean, standard deviation, n = 34) for florfenicol.

Variable	Units	Mean	SD
**C_max_**	mg/L	3.04	1.82
**AUC**	h*mg/L	53.7	9.32
**AUMC**	h*h*mg/L	650	282
**T_max_**	h	1.94	0.87
**Cl/F**	L/h/kg	0.23	0.04
**T1/2**	h	11.0	9.02

Pharmacokinetic variables determined by non-compartmental analysis for florfenicol administered intramuscularly at a dose rate of 15mg/kg; data for two bioequivalent products (Nuflor and Norfenicol). C_max_ = maximum concentration; AUC = area under plasma concentration-time curve; AUMC = area under the first moment curve; T_max_ = time to reach maximum concentration; Cl/F = drug clearance scaled by bioavailability; T_1/2_ = terminal half-life. Values are geometric means except for T_max_ (arithmetic mean) and T_1/2_ (harmonic mean).

### PK/PD integration

PK/PD integration established the parameters C_max_/MIC, T>MIC and AUC/MIC; however, for ease of interpretation, the latter is presented as concentration averages (C_av_) over three time periods (0–48, 0–24 and 24–48 h) (Table 2). Integrated values were numerically greater in serum than broth for *P*. *multocida*, with C_av0-24_/MIC being 3.78 in broth and 5.29 in serum, and for *A*. *pleuropneumoniae* data were very similar between the two media, C_av0-24_/MIC being 4.18 and 4.07. Differences were not statistically significant. For both *A*. *pleuropneumoniae* and *P*. *multocida*, the plasma concentration remained higher than MIC for approximately 36 h.

**Table 2:** Integration of pharmacokinetic (*in vivo* plasma concentration) and pharmacodynamic (MIC determined in broth and serum) variables for florfenicol (mean and standard deviation).

Organism	Parameter	Units	Broth		Serum	
			Mean	SD	Mean	SD
***Pasteurella multocida***	C_av0-48_/MIC		2.65	0.54	3.71	0.76
	C_av0-24_/MIC		3.78	1.30	5.29	1.83
	C_av24-48_/MIC		1.69	0.72	2.37	1.01
	C_max_/MIC		7.36	6.70	10.31	9.38
	T>MIC	h	35.6	18.3	41.4	22.5
***Actinobacillus pleuropneumoniae***	C_av0-48_/MIC		2.93	0.60	2.85	0.58
	C_av0-24_/MIC		4.18	1.44	4.07	1.40
	C_av24-48_/MIC		1.87	0.80	1.82	0.78
	C_max_/MIC		8.14	7.40	7.93	7.21
	T>MIC	h	37.3	19.6	37.1	19.2

C_av_ = average plasma concentration (μg/mL) for time periods 0–48, 0–24 and 24–48 h. C_max_ = maximum plasma concentration (μg/mL); T>MIC = Time for which plasma concentration exceeded MIC (h). Individual animal C_av_ and C_max_ obtained from 34 pigs divided by mean MICs for six isolates of each species measured in broth and serum.

Integration of PK and PD data, using the same PK indices and MPC, are presented in Table 3. All MPC ratios were much lower than the MIC ratios, as broth MPCs (means for 6 isolates of each species) were 10.3-fold and 13.4-fold greater than broth MICs for *A*. *pleuropneumoniae* and *P*. *multocida*, respectively. Mean C_max_/MPC ratios were less than 1.0 and C_av0-48_/MPC ratios were in the range 0.20–0.29:1 for both broth and serum and both species, so that at no time did T>MIC exceed zero.

**Table 3:** Integration of pharmacokinetic (*in vivo* plasma concentration) and pharmacodynamic (MPC determined in broth and serum) variables for florfenicol (mean and standard deviation).

Organism	Parameter	Units	Broth		Serum	
			Mean	SD	Mean	SD
***Pasteurella multocida***	C_av0-48_/MPC		0.20	0.04	0.27	0.06
	C_av0-24_/MPC		0.28	0.10	0.39	0.13
	C_av24-48_/MPC		0.13	0.05	0.17	0.07
	C_max_/MPC		0.55	0.50	0.76	0.69
	T>MPC	h	0		0	
***Actinobacillus pleuropneumoniae***	C_av0-48_/MPC		0.29	0.06	0.23	0.05
	C_av0-24_/MPC		0.41	0.14	0.32	0.11
	C_av24-48_/MPC		0.18	0.08	0.15	0.06
	C_max_/MPC		0.81	0.73	0.63	0.58
	T>MPC	h	0		0	

C_av_ = average plasma concentration (μg/mL) for time periods 0–48, 0–24 and 24–48 h. C_max_ = maximum plasma concentration (μg/mL); T>MPC = Time for which plasma concentration exceeded MPC (h). Individual animal C_av_ and C_max_ obtained from 34 pigs divided by mean MPCs for six isolates of each species measured in broth and serum.

### PK/PD breakpoints

Eight florfenicol concentrations, as multiples of MIC, ranging from 0.25 to 8xMIC, were used in time-kill studies for six isolates each of *A*. *pleuropneumoniae* and *P*. *multocida* for 24 h incubation periods. Breakpoint values of AUC_24h_/MIC producing three levels of growth inhibition are presented in Tables 4 (*P*. *multocida*) and 5 (*A*. *pleuropneumoniae*). Inter-isolate variability was small for both serum and broth values for *P*. *multocida* at each level of kill. Variability was likewise low for broth *A*. *pleuropneumoniae* breakpoints but inter-isolate variability was greater at all levels of kill for serum *A*. *pleuropneumoniae* breakpoints. The slopes of the curve were similar in serum and broth for both organisms: 3.10 and 3.63 for *P*. *multocida* in broth and serum, respectively, and, 3.06 and 3.62 for *A*. *pleuropneumoniae*.

**Table 4:** PK/PD modelling for *P*. *multocida* from time-kill curves (means and standard deviation, n = 6).

Parameter (units)	Broth		Serum	
	Mean	SD	Mean	SD
Log E_0_ (CFU/mL)	2.50	0.36	2.43	0.50
Log E_max_ (CFU/mL)	-7.02	0.70	-6.39	1.51
Log E_max_—Log E_0_ (CFU/mL)	-9.52	0.34	-8.83	1.00
Gamma	3.10	0.50	3.63	1.23
AUC_24h_/MIC for bacteriostatic action (h)	25.7	2.11	24.2	2.27
AUC_24h_/MIC_50_ (h)	35.7	2.77	32.3	3.13
AUC_24h_/MIC for bactericidal action (h)	40.2	3.39	37.3	4.00
AUC_24h_/MIC for 4 log_10_ reduction (h)	47.0	4.90	43.9	5.69

E0 = difference in number of organisms (CFU/mL) in control sample in absence of drug between time 0 and 24 h; E_max_ = difference in number of organisms (CFU/mL) in presence of florfenicol between time 0 and 24 h; AUC_24h_/MIC_50_ = concentration producing reduction in count to 50% of the E_max_; Gamma = slope of the curve; detection limit = 33 CFU/mL.

**Table 5:** PK/PD modelling for *A*. *pleuropneumoniae* from time-kill curves (mean, and standard deviation, n = 6).

Parameter (units)	Broth		Serum	
	Mean	SD	Mean	SD
Log E_0_ (CFU/mL)	2.80	0.09	2.46	0.75
Log E_max_ (CFU/mL)	-5.30	0.44	-5.29	1.48
Log E_max_—Log E_0_ (CFU/mL)	-8.10	0.35	-7.75	0.73
Gamma	3.06	1.11	3.62	1.66
AUC_24h_/MIC for bacteriostatic action (h)	24.6	1.83	30.1	13.7
AUC_24h_/MIC_50_(h)	31.1	1.38	42.1	14.5
AUC_24h_/MIC for bactericidal action (h)	43.8	5.59	58.4	19.2
AUC_24h_/MIC for 4 log_10_ reduction (h)	58.6	9.87	79.6	23.6

E0 = difference in number of organisms (CFU/mL) in control sample in absence of drug between time 0 and 24 h; E_max_ = difference in number of organisms (CFU/mL) in presence of florfenicol between time 0 and 24 h; AUC_24h_/MIC_50_ = concentration producing reduction in count to 50% of the E_max_; Gamma = slope of the curve; detection limit = 33 CFU/mL.

Dividing the AUC_24h_/MIC breakpoint ratios by 24 yields the concentrations, as MIC multiples, producing bacteriostatic, bactericidal and 4log_10_ reductions in count; these are K_PD_ values. Numerically, they were 1.07, 1.67 and 1.96 for *P*. *multocida* in broth and 1.01, 1.55 and 1.83 for this organism in serum. Corresponding values for *A*. *pleuropneumoniae* were 1.03, 1.82 and 2.44 (broth) and 1.25, 2.43 and 3.32 (serum).

### Determination of average dose at steady state

Based on means of individual animal clearances scaled by bioavailability and literature values for MIC_90_ [17], calculated daily doses for bactericidal activity were 7.77 mg/kg for *P*. *multocida* and 40.0 mg/kg for *A*. *pleuropneumoniae* (Table 6). Based on the worst case scenario, calculated using the highest single recorded clearance rate (Cl/F = 0.39 L/h/kg), the bactericidal doses were 12.3 mg/kg for *P*. *multocida* and 63.4 mg/kg for *A*. *pleuropneumoniae*.

**Table 6:** Predicted daily doses calculated by deterministic approach.

Predicted daily doses (mg/kg)		
	*P*. *multocida*	*A*. *pleuropneumoniae*
Bacteriostatic	5.05	20.6
Bactericidal	7.77	40.0
4log_10_ count reduction	9.15	54.5

MIC_90_ for *A*. *pleuropneumoniae* and *P*. *multocida* 0.5μg/mL for both organisms (de Jong et al., 2014).

### Dose determination by Monte Carlo simulation

Monte Carlo simulations were conducted using the full distributions of: (1) 34 plasma clearances scaled by bioavailability; (2) AUC_24h_/MIC breakpoints for three levels of growth inhibition; and (3) the MIC distributions reported by de Jong et al. [17], each in proportion to its incidence. Once daily doses at steady state for *P*. *multocida* and *A*. *pleuropneumoniae*, achieving a 3log_10_ decrease in count with 90% TAR were 9.58 mg/kg and 35.2 mg/kg, respectively (Table 7). Lower daily dosages were obtained for a bactericidal level of kill and 50% TAR; these were 6.50 and 21.1 mg/kg, respectively, for *P*. *multocida* and *A*. *pleuropneumoniae*.

**Table 7:** Predicted daily doses at steady state.

	Predicted daily doses (mg/kg)	Target Attainment Rate	
		50%	90%
***P*. *multocida***	Bacteriostatic	4.23	6.22
	Bactericidal	6.50	9.58
	4 log_10_ reduction	7.66	11.3
***A. pleuropneumoniae***	Bacteriostatic	10.9	18.2
	Bactericidal	21.1	35.2
	4 log_10_ reduction	28.8	48.0

Monte Carlo simulations to achieve 50 and 90% target attainment rate dosages at steady state for three levels of bacterial kill.

Table 8 indicates the single doses required for three levels of growth inhibition and durations of action of 24, 48 and 72 h. For a bacteriostatic action and 90% TAR, dosages for 24 h duration were 11.7 mg/kg (*P*. *multocida*) and 33.4 mg/kg (*A*. *pleuropneumoniae*). Higher dosages were predicted for action durations of 48 and 72 h. For example, dosages for a 90% TAR and bactericidal action over 48 h were 22.2 mg/kg (*P*. *multocida*) and 86.6 mg/kg (*A*. *pleuropneumoniae*).

**Table 8:** Single doses for 24, 48 and 72 h durations of activity.

Dose duration	Level of bacterial kill	Target Attainment Rate			
		*P*. *multocida*		*A*. *pleuropneumoniae*	
		50%	90%	50%	90%
**0-24h**	Bacteriostatic	6.93	11.7	17.7	33.4
	Bactericidal	10.7	18.0	34.2	64.6
	4 log_10_ reduction	12.6	21.1	46.7	88.2
**0-48h**	Bacteriostatic	10.8	14.4	25.0	44.7
	Bactericidal	16.6	22.1	48.5	86.6
	4 log_10_ reduction	19.5	26.1	66.1	118.1
**0-72h**	Bacteriostatic	14.8	20.0	34.6	61.6
	Bactericidal	22.8	30.8	67.1	119.4
	4 log_10_ reduction	26.9	36.3	91.5	162.9

Monte Carlo Simulations to achieve 50 and 90% target attainment rate dosages for three levels of bacterial kill and three action durations

## Discussion

### Pharmacokinetics

The aim of this study was to predict doses for florfenicol for each of the pig pneumonia pathogens, *P*. *multocida* and *A*. *pleuropneumoniae*, based on principles of PK/PD integration and modelling. The plasma concentration-time data for florfenicol were obtained for 34 healthy animals, for two products (n = 17 per product) with the same dose rate, previously shown to be bioequivalent and therefore accepted by regulatory authorities to be therapeutically equivalent from both efficacy and safety perspectives. The animals were of a single breed and similar age. In extending the present findings, it will, in future studies, be appropriate to obtain data from greater animal numbers of both sexes and differing ages and breeds and in diseased as well as healthy pigs. Thus, population PK data, derived from field studies, can be used to determine the impact of disease on clearance and bioavailability, Cl/F being a major determinant of outcome of dosage predictions of the kind reported in this paper. Nevertheless, the more limited and less variable PK data used in this study illustrates the principles of using Monte Carlo simulation to predict TAR dosages.

### Pharmacodynamics

An important consideration is whether the small number of isolates used in PK/PD integration and modelling approaches in this study, six for each of two pathogens, are representative of the much larger number of wild type isolates of these organisms present in nature in differing geographical locations Clearly, they comprise a small sample only but, nevertheless, it may be noted that: (1) MICs and time-kill curves were determined not only in broth but also in serum (possibly a more appropriate growth medium than artificial broths, as far as closeness to clinical conditions is concerned, serum of the target species being similar to although not identical with the biophase composition—for these pathogens, pulmonary epithelial lining fluid); (2) inaccuracy of individual isolate potency estimates was reduced from potentially up to 100% (when doubling dilutions are used) to up to 20% by the use of five overlapping sets of 2-fold dilutions and also by conducting each estimation in triplicate; (3) MICs were determined with a high starting inoculum count of 10^7^ CFU/mL, to reflect moderate to severe clinical disease clinically; (4) mean broth MICs in this study were 0.38 μg/mL (*A*. *pleuropneumoniae*) and 0.42 μg/mL (*P*. *multocida*) and these may be compared with broth MIC_90_ values of 0.50 μg/mL for both organisms reported by de Jong et al. (2014); and (5) the present data were supported by an additional potency index, namely MPC and the proportional increase in potency compared to MIC was similar for determinations in broth and serum. These considerations give confidence in the present data, in respect of being representative of the much larger number of wild type isolates.

### PK/PD integration

The PK/PD integration approach adopted in this study provides a useful starting point for evaluating efficacy of dosage for the pig pneumonia pathogens, *P*. *multocida* and *A*. *pleuropneumoniae*. Thus, average florfenicol concentrations in plasma were at least 2.5-fold greater than MICs for 48 h after dosing and T>MIC was in the range 35.5–41.3 h for both broth and serum values. One might therefore predict some efficacy in clinical subjects, especially if pathogen load in the biophase is low to moderate, as MICs of florfenicol have been shown to be inoculum size dependent [16]. However, the PK/PD index and its numerical value that best correlate with bacteriological cure are not known for this drug and can only be surmised, in the absence of high quality clinical data.

As MPCs were at least 10-fold greater than corresponding MICs for both growth matrices and both pathogens, the integration of PK and PD data indicated that concentrations could not be achieved, with clinical dose rates in healthy pigs, which would ensure the eradication of the least sensitive sub-population in a given colony. In fact, even at C_max_ the concentration ratios to MPC for serum were 0.76:1 (*P*. *multocida*) and 0.63:1 (*A*. *pleuropneumoniae*). Hence, at no time was T>MIC above zero.

Whilst serum is not identical to extravascular infection site fluids, it is likely to be closer to the biophase than broths in chemical composition and indeed in respect of immunological components also [16]. A comparison of broth MICs with potency determined in biological fluids is therefore relevant to PK/PD breakpoint estimation, when the aim is optimal dose prediction for clinical use. For some drugs and pathogens, calculation of a scaling factor to bridge between broth and serum MICs may be warranted [16].

### PK/PD modelling and breakpoint determination

The advantage of PK/PD modelling over PK/PD integration is that it yields breakpoint values for a particular drug against a particular pathogen and, moreover, it defines the whole sweep of the concentration-effect relationship, so that any pre-determined level of activity, ranging from bacteriostasis to virtual eradication, indicated by AUC_24h_/MIC, can be determined.

For *P*. *multocida* a bacteriostatic effect was obtained in growth inhibition studies with multiples of MIC of 1.07 and 1.01, respectively, for broth and serum. Corresponding values for a bactericidal action were 1.68 and 1.55. Similarly, the breakpoint PK/PD index, AUC_24h_/MIC, for six-calf isolates revealed a bacteriostatic effect with multiples of MIC of 0.89 and 0.95, respectively, for broth and serum [18]. Corresponding values for a bactericidal action were 1.26 and 1.34 [18], suggesting, at most, small differences in breakpoints between calf and pig *P*. *multocida* isolates.

### Dosage prediction

In considering predictions of dosage, based on the pharmacokinetics of florfenicol in healthy pigs and in vitro estimates of potency, it is important to recognise that: (1) pharmacokinetic profiles might well differ in pigs of differing age and breed and between healthy and diseased animals and ideally population pharmacokinetic data should be used in PK/PD modelling studies; (2) potency as measured by MICs may not be identical in serum as in biophase fluids; (3) drug concentrations in the biophase may not equal free drug concentrations in serum even at steady state; and (4) all dosage estimates require confirmation in animal disease models and then in clinical trials. Moreover, previous researchers have shown that maintaining antibacterial concentrations above the MIC, when the inoculum size increases with high density populations, such as those likely in acute infection, can produce growth and selective amplification of mutant bacteria. For this reason, maintaining drug concentration greater than MPC can block the mutant cell growth. However, in the case of florfenicol dosed at 15 mg/kg at 48 h in pigs, MPC values could not be achieved. These considerations indicate that this dosage could selectively enrich susceptible mutant sub-populations with MPC values close to 4–5 mg/L or higher. On the other hand, after prediction of dosage at steady state, single 90%TAR doses close to 14.4 to 26.1 mg/kg or higher were obtained. In this situation, these doses could be more effective than the standard dose of 15 mg/kg at 48 h intervals.

Initially, a deterministic approach to dosage prediction was undertaken. It provides a useful estimate of once daily doses at steady state, based on MIC_90_ as a single value and average values for other variables, but it does not take account either of variability in or incidence of each input variable. Nevertheless, it does provide an initial evaluation of data prior to further Monte Carlo simulations to estimate population doses for each selected TAR, which is a dose encompassing a given percentile of the target population, for example 50 or 90% and for three levels of bacterial kill. Moreover, Monte Carlo simulations predict doses, allowing for incidence within MIC distributions and encompassing best, worst and all intermediate case scenarios for distributions of Cl/F and AUC_24h_/MIC ratios. Additionally, Monte Carlo simulations were used to predict optimal daily TAR doses for maintaining steady state conditions as well as for TAR single doses over 24, 48 or 72 h time periods, which is for a single or a loading dose, in the latter case possibly to be repeated after a selected time interval.

For single doses and a duration of action of 48 h, 90% TAR doses for *P*. *multocida* were 14.4 mg/kg (bacteriostasis), 22.2 mg/kg (bactericidal action) and 26.1 mg/kg (4log_10_ reduction in bacterial count). Comparative values for *A*. *pleuropneumoniae* were higher, 44.7, 86.6 and 118.4 mg/kg. With the exception of the bacteriostatic dose for *P*. *multocida*, these predicted doses are higher than the currently recommended dose of 15 mg/kg by intramuscular injection at 48 h intervals. On the other hand, once daily 90% TAR doses predicted for florfenicol and *P*. *multocida* isolates were 4.23, 6.50 and 7.66 mg/kg at the three levels of kill. Corresponding 90% TAR values for bovine derived *P*. *multocida* isolates were 1.23, 2.87 and 4.18 mg/kg [19]. The differences in dose between these animal species could be due to a number of factors, including serum protein binding, Cl/F, PK/PD breakpoints and MIC distribution differences between the calf and pig isolates. In fact, values of Cl/F were almost identical (228 and 223 mL/h/kg, in calf and pig, respectively, [19] and this study) whereas AUC_24h_/MIC breakpoints were lower for the calf (7.6, 18 and 25 h) than for the pig (24, 37 and 43 h) isolates of *P*. *multocida*. If this calf/pig isolate difference in breakpoint values is allowed for, the adjusted calf doses of 3.87, 5.89 and 7.19 mg/kg would be very similar to those calculated for the pig. Another difference between calf and pig, however, is in concentration of free (and microbiologically active) drug fraction in serum; this was 82% in the calf and 35% in the pig and this 2.3-fold difference might also, even considered alone, account largely for the differences in predicted doses for the two species [16].

The Monte Carlo simulation basis for predicting dosage for clinical use has the advantage of taking account of all important PK and PD variables impinging on bacteriological outcome. Furthermore, basing potency estimates on serum as a growth matrix may have greater relevance to *in vivo* conditions than artificial broths, whilst recognising that serum will be similar, but not identical, in composition to the biophase at infection sites. Moreover, the PK/PD Monte Carlo approach has established three levels of bacterial kill (bacteriostatic, -cidal and virtual eradication) for two pig pneumonia microorganisms and pre-selected TARs. It also allows the TAR percentage to be set, for example at 90%, but other TAR percentages can be determined and applied, and the simulation models all data in relation to incidence. Finally, in this study several steps were taken to minimise avoidable errors, including conducting breakpoint determinations in triplicate for each isolate.

Despite these clear advantages, there are inevitable limitations to study methodology and conclusions reached. The MIC distribution data were extracted from the literature, and the number of isolates was moderate but limited (229 for *P*. *multocida* and 220 for *A*. *pleuropneumoniae*). Moreover, they were obtained from a restricted geographical location (Europe). Whilst the inter-isolate variability in PK/PD breakpoint values was small to moderate, estimates were based on only six isolates for each species. The time-kill studies used fixed drug concentrations (eight multiples of MIC) for a fixed time period (24 h). In clinical use, on the other hand, plasma drug concentrations would first increase and then decrease after intramuscular dosing, exposing organisms to a continuously variable concentration. At present, information on MPC and, related to this, Mutant Selection Window (MSW), for florfenicol and the two pathogens investigated in this study is limited. We are not aware of other published data on MPC and MSW for this drug and these organisms. Clinical breakpoint and optimal PK/PD relations for MSW and MPC are not well known in comparison to MIC and little information is available on MPC distribution. Drug concentrations in the biophase (in this case pulmonary epithelial lining fluid) may not be identical to free drug concentrations in serum, even at steady state.

In future studies, these concerns could be addressed by increasing numbers of isolates surveyed in field distribution studies and used in experimental PK/PD modelling studies to establish breakpoint values. Exposure of organisms to varying drug concentrations could be addressed by use of *in vitro* pump methods to simulate *in vivo* patterns of change in concentration with time [20]. This study was confined to two pig pneumonia pathogens. In future studies the approach could be extended to other pathogens. Additional data on MPC and MSW for florfenicol and pig pathogens is clearly required. Their influence on dosing strategies and the importance of limiting the development of resistance in food producing animals remains to be determined. Future studies might also be based on PK/PD modelling approaches based on drug concentrations in the biophase. Finally, the methodology does not incorporate the contribution to pathogen elimination by the body's natural defence mechanisms in clinical subjects, nor does it allow for additional potentially beneficial properties of antimicrobial drugs, such as immunomodulatory and anti-inflammatory actions.

### Conclusions

Predicted doses for florfenicol for treatment of respiratory tract infections in the pig, caused by *P*. *multocida* or *A*. *pleuropneumoniae*, were determined by integrating and modelling PK and PD data obtained experimentally, together with literature data on MIC distributions. The findings illustrate the value and principle of using Monte Carlo simulations for determination of optimal doses. These methods provide a powerful tool to optimise doses for subsequent evaluation in disease models and clinical trials.
